# Molecular Aspects of Hypoxic Stress Effects in Chronic Ethanol Exposure of Neuronal Cells

**DOI:** 10.3390/cimb45020107

**Published:** 2023-02-16

**Authors:** Simona Isabelle Stoica, Gelu Onose, Ioana Madalina Pitica, Ana Iulia Neagu, Gabriela Ion, Lilia Matei, Laura Denisa Dragu, Lacramioara-Elena Radu, Mihaela Chivu-Economescu, Laura Georgiana Necula, Aurelian Anghelescu, Carmen Cristina Diaconu, Constantin Munteanu, Coralia Bleotu

**Affiliations:** 1 Faculty of Medicine, University of Medicine and Pharmacy “Carol Davila” (UMPCD), 020022 Bucharest, Romania; 2 Teaching Emergency Hospital “Bagdasar-Arseni” (TEHBA), 041915 Bucharest, Romania; 3 Stefan S. Nicolau Institute of Virology, 285 Mihai Bravu Avenue, 030304 Bucharest, Romania; 4 Grigore T. Popa University of Medicine and Pharmacy of Iași, 700454 Iași, Romania; 5 Romanian Academy of Scientists, 54 Spl. Independenței Str., District 5, 050085 Bucharest, Romania; 6 Faculty of Biology, University of Bucharest, 1-3 Aleea Portocalelor Str., District 5, 060101 Bucharest, Romania

**Keywords:** hypoxic stress effect, chronic ethanol exposure, neuronal cells, neurorehabilitation

## Abstract

Experimental models of a clinical, pathophysiological context are used to understand molecular mechanisms and develop novel therapies. Previous studies revealed better outcomes for spinal cord injury chronic ethanol-consuming patients. This study evaluated cellular and molecular changes in a model mimicking spinal cord injury (hypoxic stress induced by treatment with deferoxamine or cobalt chloride) in chronic ethanol-consuming patients (ethanol-exposed neural cultures (SK-N-SH)) in order to explain the clinical paradigm of better outcomes for spinal cord injury chronic ethanol-consuming patients. The results show that long-term ethanol exposure has a cytotoxic effect, inducing apoptosis. At 24 h after the induction of hypoxic stress (by deferoxamine or cobalt chloride treatments), reduced ROS in long-term ethanol-exposed SK-N-SH cells was observed, which might be due to an adaptation to stressful conditions. In addition, the HIF-1α protein level was increased after hypoxic treatment of long-term ethanol-exposed cells, inducing fluctuations in its target metabolic enzymes proportionally with treatment intensity. The wound healing assay demonstrated that the cells recovered after stress conditions, showing that the ethanol-exposed cells that passed the acute step had the same proliferation profile as the cells unexposed to ethanol. Deferoxamine-treated cells displayed higher proliferative activity than the control cells in the proliferation–migration assay, emphasizing the neuroprotective effect. Cells have overcome the critical point of the alcohol-induced traumatic impact and adapted to ethanol (a chronic phenomenon), sustaining the regeneration process. However, further experiments are needed to ensure recovery efficiency is more effective in chronic ethanol exposure.

## 1. Introduction

Spinal cord injuries (SCI) comprise a particular category of trauma based on their severity and complex clinical management. The failure of SCI treatment and incomplete recovery is due to a poor understanding of their intricate characteristics, abundant inconsistencies, and complex pathophysiologic consequences post-SCI [1]. Post-traumatic SCI changes comprise the acute phase (up to 48 h post-SCI), the subacute phase (between 2 days and two weeks post-trauma), and the chronic one (following the subacute phase) [2,3]. The acute phase is divided into an immediate phase (in the first 2 h) and an early acute phase (from 2 to 48 h post-trauma). It has been shown that there are axonal and gray matter damage, bleeding, local ischemia, and neuronal death in the immediate acute phase [4]. At this stage, the microglia cells are activated with the release of tumor necrosis factor-alpha (TNF-α) and interleukin-1β (IL-1β) (even from the first minutes after the injury) and with cytotoxic amounts of glutamate production [5].

TNF-α mRNA expression has been detected in non-neuronal cells from the injured area starting with the first post-injury hour [6]. In the early acute phase, intramedullary bleeding continues, leading to local edema and inflammation with the release of free radicals, local ionic disturbances [7], and glutamate-dependent excitotoxicity. Therefore, the process of neuronal death continues, and ischemic changes affect neurons and glial cells. There is an interruption in the continuity of the blood–spinal cord barrier in the first 24 h, with an intra-tissular neuraxial accumulation of nitric oxide, histamine, matrix metalloproteinases, and elastase. Neuronal death and bleeding at the injury site are associated with axonal destruction and disruption of neuron membrane continuity [8].

Some factors, such as pre-injury alcohol or drug use, have been recognized to influence the proper rehabilitation after SCI [9]. Nevertheless, our clinical experience has shown a particular SCI situation in chronic alcohol-consuming patients compared to those who did not consume excessive alcohol until the initial post-traumatic moment [10]. Thus, a statistically significant difference was observed between the lowest degrees of post-traumatic distress (sensitive and motor) in chronic alcohol-consuming patients compared to those who did not abuse ethanol in their diet [10]. There was also a statistically significant difference in the clinical condition of patients, consumers or non-consumers of ethanol, at discharge, compared to the time of their admission to the neuromuscular division, with superior improvement in neurological deficits (sensory and motor) in patients with a history of chronic ethanol consumption, compared to patients with a balanced diet in terms of alcohol consumption [10]. All these intriguing clinical findings led us to look for the potential cause of the differences in the condition and evolution of the patients by studying the molecular basis of the process behind the clinical outcome using a cellular model of SCI.

Thus, we aimed to evaluate the neural cells exposed in vitro to ethanol and experimental hypoxic conditions as a model of events after spinal cord injury. As hypoxia inducers, we used: deferoxamine (DFX) and cobalt chloride (CoCl_2_), substances often used experimentally to simulate hypoxic conditions at the cellular level [11,12,13,14,15,16,17]. This article reports overcoming some hypoxia effects after cellular adaptations developed in long-term ethanol-exposed neural cultures.

## 2. Results

### 2.1. Evaluation of the Viability of SK-N-SH and Setting Ethanol Long-Term Exposure Concentrations

**Figure 1:**
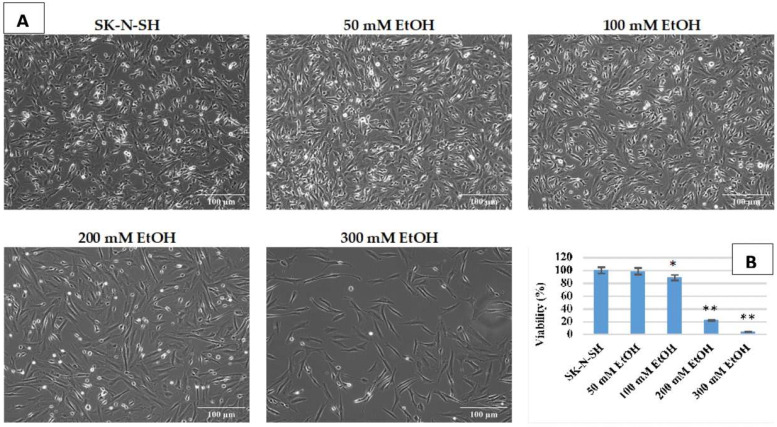
Phase contrast images, obtained using 10× objective, presenting the morphology (**A**) and viability (**B**) of SK-N-SH cells exposed at different ethanol concentrations (*p*-value < 0.05 was considered statistically significant, * *p* < 0.05, ** *p* < 0.005).

To establish the experimental conditions of chronic ethanol exposure, SK-N-SH cells were first treated daily for 7 days with the following increasing ethanol concentrations: 50 mM, 100 mM, 200 mM, and 300 mM. As shown in Figure 1, concentrations higher than 100 mM ethanol lead to extensive cell death. After treatment with 300 mM ethanol, viability was only 4% of the untreated control. A concentration of 50 mM ethanol does not affect cell viability after 7 days (98.7%) and has therefore been selected for chronic—long-term—ethanol exposure, respectively, of more than 9 weeks.

### 2.2. Evaluation of the Effect of Hypoxia Inducers

**Figure 2:**
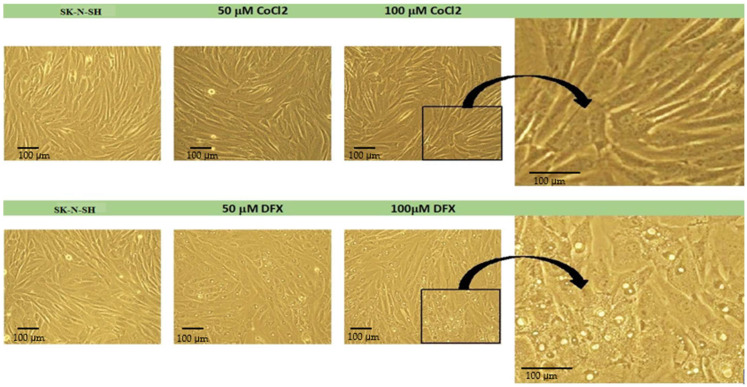
The morphology of SK-N-SH cells treated with CoCl_2_ or DFX (phase contrast images obtained using 20× objective). The black border shows the region presented on the right (magnified images).

CoCl_2_ and DFX at 50 μM and 100 μM concentrations were used as hypoxia inducers [18]. Cells were treated for 24 h. As shown in Figure 2, treatment with CoCl_2_ does not affect cell morphology, whereas in DFX treatment, morphological changes occur with the appearance of inclusions. These inclusions most likely signify impairments of the cellular metabolism connected with mitochondrial dysfunctions as DFX treatment causes acute mitochondrial swelling in a concentration-dependent manner [19].

### 2.3. Evaluation of the Effect of Hypoxia Inducers on the Viability of Long-Term Ethanol-Exposed SK-N-SH Cells

**Figure 3:**
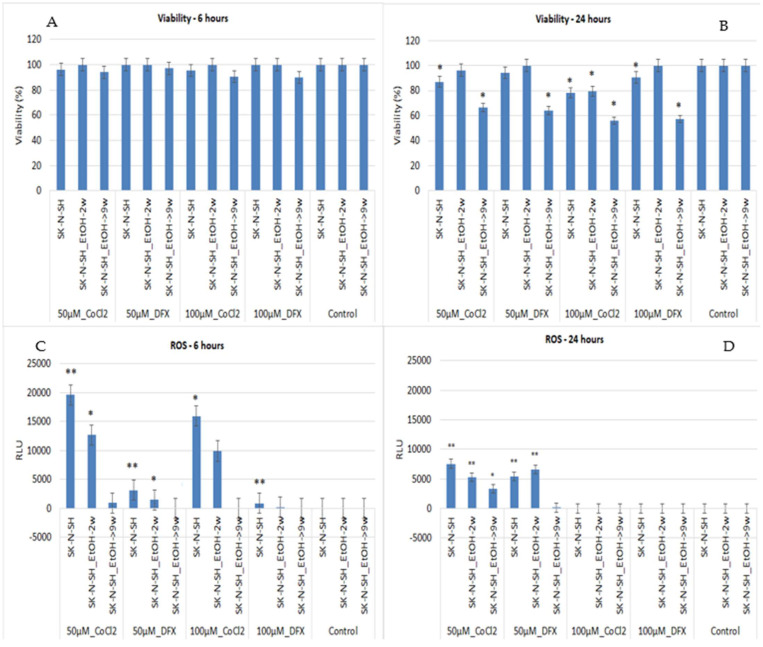
Quantification of cell viability and ROS release at 6 and 24 h in ethanol-exposed and non-exposed SK-N-SH cells treated with different concentrations of DFX and CoCl_2_. A *p*-value < 0.05 was considered statistically significant, * *p* < 0.05, ** *p* < 0.005.

The treatment of long-term ethanol-exposed cells with hypoxia inducers for 6 h does not affect cell viability (Figure 3A). Moreover, the viability of SK-N-SH_EtOH-2w cells is not affected after 24 h of 50 µM CoCl_2_ or DFX treatment (Figure 3B). The same treatment (50 µM CoCl_2_) applied to SK-N-SH_EtOH-9w induces a significant drop in cell viability compared to SK-N-SH_EtOH-9w untreated with hypoxic agents. The 24 h treatment with 100 µM CoCl_2_ decreased the viability of SK-N-SH cells compared to the untreated cells. SK-N-SH_EtOH-2w cells treated with 100 µM CoCl_2_ have the same viability as SK-N-SH 100 µM CoCl_2_ treated cells. However, 100 µM CoCl_2_ applied on SK-N-SH_EtOH-9w significantly drops the viability compared to controls (Figure 3B).

Regarding the DFX effect, 24 h of treatment with 100 µM DFX did not change the viability of SK-N-SH_EtOH-2w cells but significantly decreased the viability of SK-N-SH_EtOH-9w compared to the control. It should be noted that the presence of ethanol for long periods induces resistance to 6 h of hypoxic treatment (Figure 3A). SK-N-SH_EtOH-2w cells show resistance even to treatment for 24 h with 100 µM DFX (Figure 3B).

### 2.4. Evaluation of the Effect of Hypoxia Inducers on ROS Release by Long-Term Ethanol-Exposed SK-N-SH Cells

The ROS evaluation after 6 h treatment of SK-N-SH neural cells (control) with CoCl_2_ induced an exacerbated increase in ROS at low concentrations (50 µM) while DFX induced only a slight increase in ROS. Similarly, the cells exposed for 2 weeks to ethanol showed the same pattern while 9w chronic ethanol treatment showed only a slight increase in ROS in the case of CoCl_2_ treatment.

At 24 h, the treatment of SK-N-SH cells with 50 µM CoCl_2_ or DFX induced higher ROS release than untreated cells. The same pattern was maintained by SK-N-SH_EtOH-2w cells following 24 h treatments with 50 µM CoCl_2_ or DFX (Figure 3D). A high concentration of DFX (100 µM), for either 6 h or 24 h treatment, did not induce ROS elevation. Chronic ethanol treatment maintains ROS levels at minimal in the presence of hypoxia inducers except when cells were treated with 50 µM CoCl_2_ for 24 h. Long-term exposure of SK-N-SH-9w cells to ethanol might induce an adaptation to stressful conditions. Moreover, when another additional stressor appears, cells can be less affected, and the cellular response to ROS production is inhibited.

### 2.5. Apoptosis Evaluation in Ethanol-Exposed and Non-Exposed Cells Treated with Hypoxic Agents

**Figure 4:**
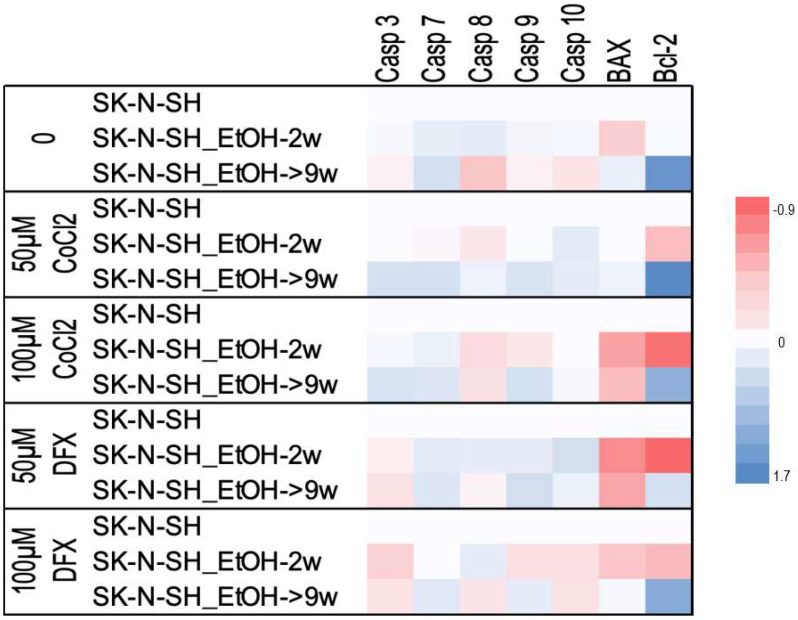
Heatmap showing the levels of apoptotic gene expression in ethanol-exposed and non-exposed cells treated with hypoxic agents. Each numerical data was graphically represented using warm colors for low-value data points and cool colors representing high-value data points.

The expression of caspases involved in both extrinsic (caspase-8 and caspase-10) and intrinsic apoptotic pathways (caspase-9) and effector caspases (caspase-7 and caspase-3) were studied. Caspases are secreted in their inactive form (zymogen or pro-caspases) and activated by the initiating factors. Cellular apoptotic modifications occur due to caspases activity, such as cytoplasmic secretions (in the extracellular environment) and chromatin condensation. Caspases-3, -6, and -7 represent the final common point of the apoptotic pathways (intrinsic and extrinsic); they act by disrupting DNA and other cellular components, reaching the final apoptotic cellular phenotype [20]. SK-N-SH_EtOH-2w cells showed a higher level of all investigated caspases than SK-N-SH cells (Figure 4). SK-N-SH_EtOH-9w cells showed a high level of caspase-7 and a downregulation of all other caspases compared to SK-N-SH cells. The treatment with hypoxic agents changed the pattern of caspases expression in long-term ethanol-exposed cells. The level of caspase-7 remained high in SK-N-SH_EtOH-9w cells regardless of the hypoxia agent used. DFX downregulated Caspase-3 in both SK-N-SH_EtOH-2w and SK-N-SH_EtOH-9w compared to non-ethanol exposed cells. SK-N-SH_EtOH-9w cells showed a high level of expression of caspase-3 induced by CoCl_2_ (Figure 4).

When we evaluated gene expression after grouping based on quantities of hypoxic agent treatment, we observed that in comparison with non-exposed to ethanol cells, treated or untreated with hypoxic agents, the Bcl-2 expression levels were increased in all cells maintained for a long time in ethanol, while the expression of Bax was decreased or only slightly increased. This could be attributed to a slight inhibition of apoptosis in long-term ethanol exposure.

The Proteome Profiler Apoptosis Assay was used to analyze the intrinsic and extrinsic apoptotic pathways in SK-N-SH_EtOH-9w. The results were expressed as a fold change of the expression in SK-N-SH_EtOH-9w cells treated with hypoxic agents relative to SK-N-SH (control cells).

**Figure 5:**
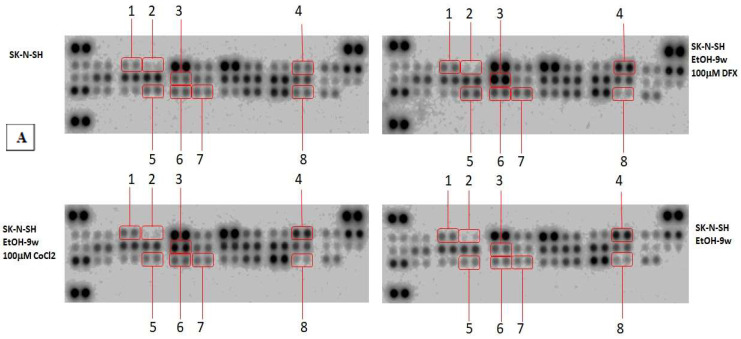

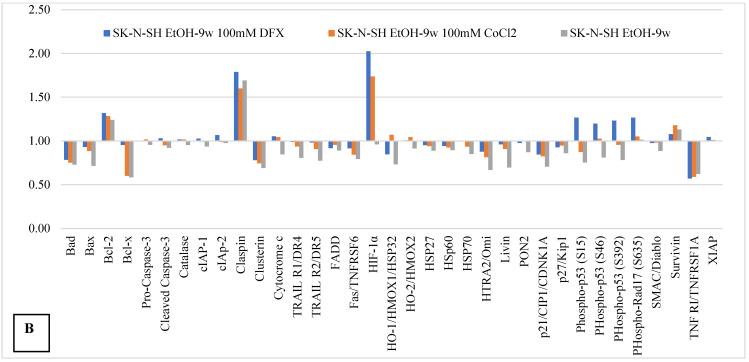
The effects of hypoxia mimetic treatment on apoptosis-related proteins in chronic ethanol-treated SK-N-SH cells. The total protein lysates (400 μg) were subjected to proteome profiler array analysis. (**A**) Dot-blot image: 1. Bcl-2; 2. Bcl-x; 3. HIF-1α; 4. Claspin; 5. Phospho-p53 (S15); 6. Phospho-p53 (S46); 7. Phospho-p53 (S392); 8. TNF RI/TNFRSF1A. (**B**) Fold change compared to ethanol-unexposed cells (logarithmic scale). The intensity of each spot was quantified using ImageJ software, and the resulted graph shows fold change of proteins measured after hypoxic treatment relative to untreated ethanol-unexposed control cells.

The expression levels of the pro-apoptotic proteins Bad and Bax and antiapoptotic mitochondrial protein Bcl-x (Figure 5) were decreased in chronic ethanol-treated SK-N-SH cells, as well as in the presence of 100 µM DFX or CoCl_2_ compared to their expression in untreated control cells. In contrast, the antiapoptotic protein Bcl-2 was increased in SK-N-SH-9w cells compared to control cells.

**Figure 6:**
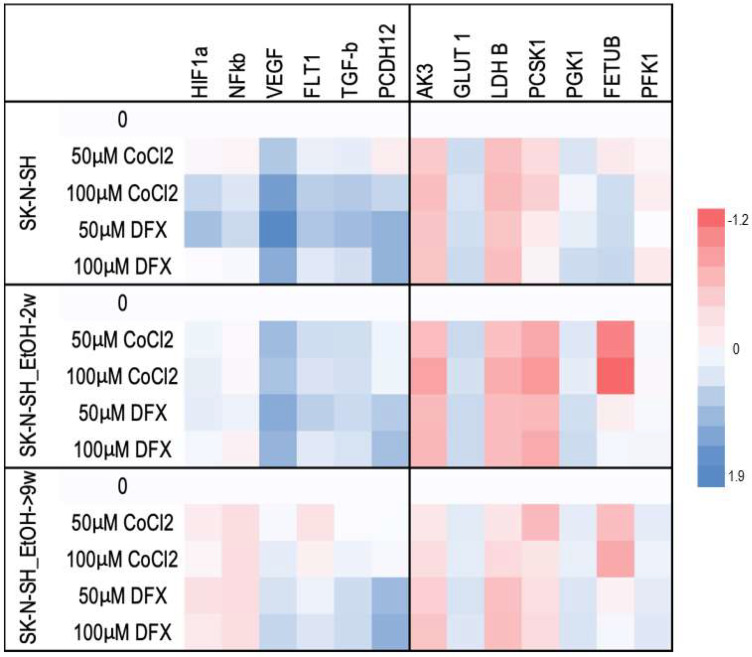
Heatmap analysis of stress and metabolic gene expression profile induced by DFX and CoCl_2_ on ethanol-exposed SK-N-SH cells. Each numerical data was graphically represented using warm colours representing low-value data points and cool colours representing high-value data points.

Apoptotic cell death receptors and adapters (TRAILR1 (DR4), TRAILR2 (DR5), FADD, Fas, and OMI/HTRA2), the inactive form of caspase-3 (pro-caspase-3) and cleaved caspase-3, as well as apoptosis inhibitory proteins (cIAP-1, cIAP-2, and Livin), a small mitochondria-derived activator of caspases/direct inhibitor of apoptosis-binding protein with low pI (SMAC/DIABLO), and cytochrome C (component element of the apoptosome), were very weakly affected by both chronic alcohol treatment and hypoxic agents. Heat shock proteins, HSP27 (with cytoprotective role against oxidative stress), HSP60 (involved in the maintaining of cellular/mitochondrial energy balance), and HSP70 (with antiapoptotic and anti-inflammatory roles) were weakly inhibited by the presence of alcohol and hypoxic treatment under the tested conditions. In addition, molecules involved in cell cycle progression, such as p21/CIP1/CDNK1A, a cell cycle inhibitory protein [21,22,23] and p27/Kip1, a multifunctional Cyclin-Dependent Kinase Inhibitor [24], were slightly inhibited by chronic ethanol-treatment. These data, together with the observation that survivin (a member of the inhibitor of apoptosis (IAP) protein family) was slightly increased, might support the idea of some protection achieved by chronic ethanol treatment [25]. Moreover, a high expression of claspin, a cell cycle regulating protein, was associated with chronic ethanol consumption. Hypoxic agents do not seem to affect claspin expression induced by long-term ethanol exposure. On the contrary, clustering, a ubiquitously expressed cell protein, was inhibited by long-term ethanol exposure, and the presence of hypoxic agents kept its expression in the same range (Figure 6).

SK-N-SH-9w cells treated with 100 µM DFX showed the activation of p53 by phosphorylation at positions S46, S392, and S15 (Figure 6). In the same manner, serine 635 phosphorylation of Rad17 was activated in SK-N-SH-9w cells treated with 100 µM DFX. TNFR-1, one of the receptors for apoptosis-inducing proteins (extrinsically), was inhibited in SK-N-SH-9w cells. Its inhibition was maintained even in additional hypoxic conditions induced by treatment with either DFX or CoCl_2_ (Figure 5).

Protein synthesis controlled by the Bax gene was inhibited following hypoxemic treatment of SK-N-SH cells (especially following treatment with DFX 100 µM), as well as in cultures chronically exposed to ethanol and subjected to added hypoxemic stress. Protein synthesis controlled by the Bcl-2 gene was inhibited by hypoxemic conditions in SK-N-SH cultures and was stimulated under conditions of chronic ethanol treatment of SK-N-SH cultures. We observe a correspondence between Bax and Bcl-2 gene expression and consequent protein synthesis.

The Bcl-x gene controls the synthesis of an anti-apoptotic protein that is inhibited in cell cultures chronically exposed to ethanol, an inhibition that is diminished under hypoxic conditions produced by treatment with deferoxamine 100 µM and cobalt chloride 100 µM. The dynamics of Bcl-x protein synthesis show how apoptotic distress (induced by chronic cellular ethanol exposure) is ameliorated under superimposed hypoxic distress, with a possible propensity for more pronounced survival.

### 2.6. Analysis of Stress Effects Induced by DFX and CoCl_2_ on Alcohol-Treated SK-N-SH Cells

To evaluate the hypoxic effect of the two hypoxia inducers (DFX and CoCl_2_), two concentrations proven to have mild cytotoxic effect after a 24 h treatment were used. We evaluated the HIF mRNA expression, an important molecule in the detection and adaptation of cells to oxygen levels and, consequently, in response to hypoxia [26]. The mRNA expression was evaluated in treated and untreated ethanol cells.

**Figure 7:**
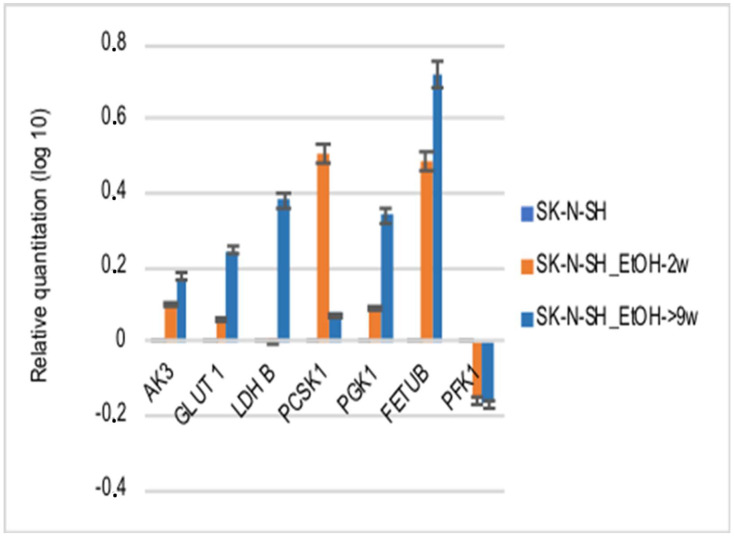
The effects of ethanol treatment on the expression of the gene that codes metabolic enzymes.

HIF-1α is one of the major regulators of the cell response to hypoxic conditions. HIF-1α protein expression increased in SK-N-SH-9w cells treated with 100 µM DFX or CoCl_2_ (Figure 6). Moreover, HIF-1α mRNA expression was evaluated (Figure 6). In cells maintained without ethanol, HIF-1α mRNA expression was induced by a lower concentration of DFX and a higher concentration of CoCl_2_. In cells maintained for 2 weeks in alcohol, HIF-1α mRNA expression was slightly increased by both hypoxia inducers. Notably, the expression of HIF-1α mRNA was decreased in the SK-N-SH-9w cells due to the action of hypoxic agents (Figure 7). Chronic ethanol treatment appears to inhibit HIF-1α gene expression, regardless of the hypoxemic agents used in the experiment. Therefore, SK-N-SH-9w cells treated with 100 µM DFX or CoCl_2_ had low HIF-1α mRNA, high HIF-1α protein, low ROS, and low Bax but high Bcl-2 levels.

For a better understanding of the chronic ethanol exposure effect on neural cells, the following HIF-1α targets were evaluated: vascular endothelial growth factor (VEGF), glucose transporter 1 (GLUT-1), adenylate kinase 3 (AK-3), aldolase A (ALD-A), phosphoglycerate kinase 1 (PGK-1), 6-phosphofructokinase, liver type (PFK-L), and lactate dehydrogenase B (LDH-A). The mRNAs of VEGF and its FLT1 receptor in SK-N-SH-9w cells followed the decrease of HIF-1α mRNA (Figure 6).

The HIF-1α target metabolic enzymes, presented in Figure 7, generally show an increased expression in both long-term ethanol-treated cell lines, SK-N-SH-2w and SK-N-SH-9w. It was observed that Adenyl kinase 3 (AK3) gene expression was stimulated in SK-N-SH cell cultures chronically exposed to ethanol, but hypoxic stress inhibited AK3 expression (Figure 7). We observed a decreasing tendency of AMP phosphorylation (directly proportional to the molar concentration) in the case of DFX treatment. At the same time, exposure to CoCl_2_ increased the phosphorylation of AMP (at a concentration of 50 µM). Still, this enzymatic process decreased its yield by increasing the concentration of CoCl_2_ in the culture medium. Most likely, the hypoxic stress causes cellular energy depletion proportional to its intensity. The glycolysis analysis included the aerobic pathway (phosphoglycerate kinase-1 PGK1), the anaerobic pathway (by phosphofructokinase-PFKL1, lactate dehydrogenase B-LDH B), the glucose transporter (GLUT1), and the protein sub-convertase/kexin type 1 (PCSK1) involved in the proteolytic activation of insulin. It has been observed that PGK1 gene expression was stimulated in SK-N-SH cell cultures treated chronically with ethanol, and hypoxic stress further increases PGK1 values. PFKL1 gene expression was inhibited by chronic ethanol treatment in SK-N-SH cell cultures, but hypoxic stress increased its expression, except after CoCl_2_ treatment. LDH B gene expression was stimulated in chronic ethanol treatment in SK-N-SH cultures but was inhibited by hypoxic stress. Intracellular glucose transport (GLUT1) was increased in chronically ethanol-treated cell cultures, and stress induced by hypoxic agents improved this process. On the contrary, PCSK1 gene expression was stimulated in ethanol-exposed SK-N-SH cell cultures, but hypoxic agents reduced PCSK1 mRNA levels. An ethanol-induced increase in fetuin B gene expression (FETU-B) had been noticed. However, in the cells not exposed to ethanol, only DFX, but not CoCl_2_ treatment, increased FETU-B. FETU-B expression was also inhibited in cells maintained in ethanol and exposed to hypoxic stress.

**Figure 8:**
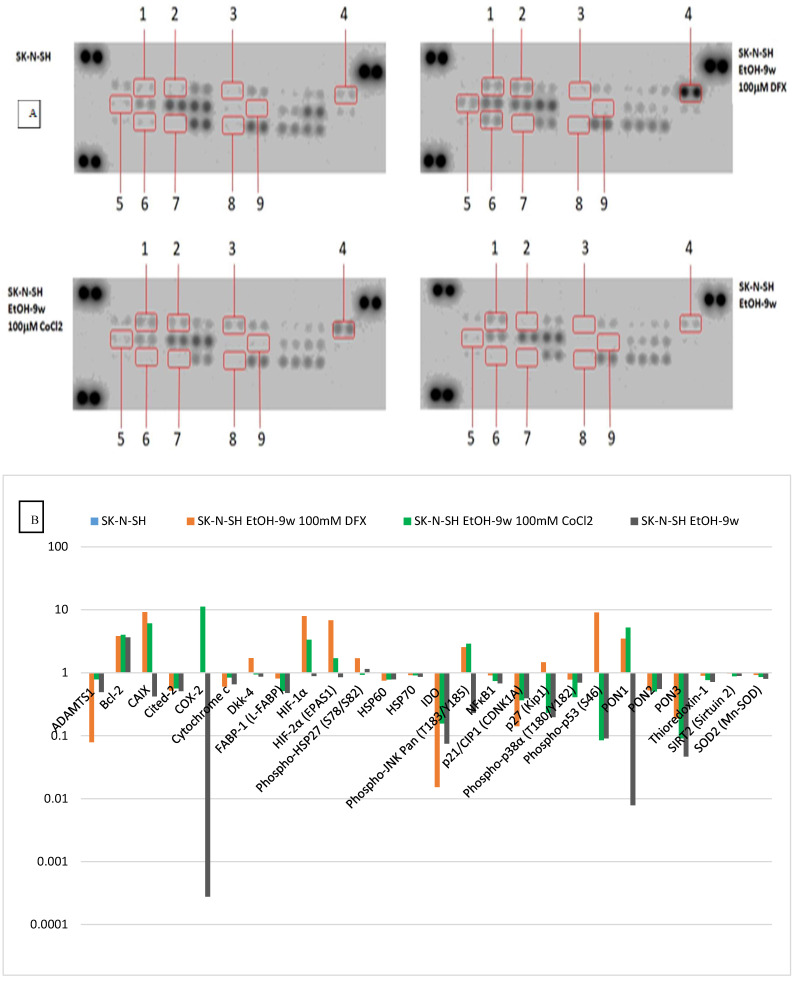
The effects of hypoxic treatment on stress-related proteins in long-term ethanol-exposed SK-N-SH cells. The total protein lysates (100 μg) were subjected to the proteome profiler stress array analysis. (**A**) Dot-blot image: 1. Bcl-2; 2. Carbonic Anhydrase IX (CA9); 3. COX-2; 4. HIF-1α; 5. HIF-2α (EPAS1); 6. Phospho-p53 (S46); 7. PON1; 8. PON3; 9. Phospho-JNK Pan (T183/Y185. (**B**) Fold change relative to control cells (unexposed to ethanol) (logarithmic scale). The intensity of each spot was quantified using ImageJ software. The resulting graph shows the fold change of proteins measured for hypoxic treatment relative to control cells maintained without ethanol and hypoxic agents.

HIF-1α and HIF-2α proteins, markers of intracellular hypoxia [26], were inhibited in the presence of ethanol but increased by treatment with hypoxic agents. Some proteins known to be affected by cellular stress showed similar dynamics in our experiments (Figure 8). Thus, the synthesis of CA IX, Phospho-JNK, HIF-1α, HIF-1β, and PON1 were stimulated in the presence of hypoxic agents, although the chronic exposure to ethanol had determined their inhibition. In chronically exposed ethanol SK-N-SH cells, a decrease in indole-2,3-dioxygenase (IDO) synthesis was observed; this inhibition was also found following hypoxia conditions (at 100 µM DFX or 100 µM CoCl_2_) favoring serotonin synthesis. Exposure of SK-N-SH cells to the chronic-ethanol treatment and hypoxic conditions negatively regulates the intrinsic apoptotic pathway by decreasing the synthesis of Cited-2 and cytochrome C (Figure 8).

**Figure 9:**
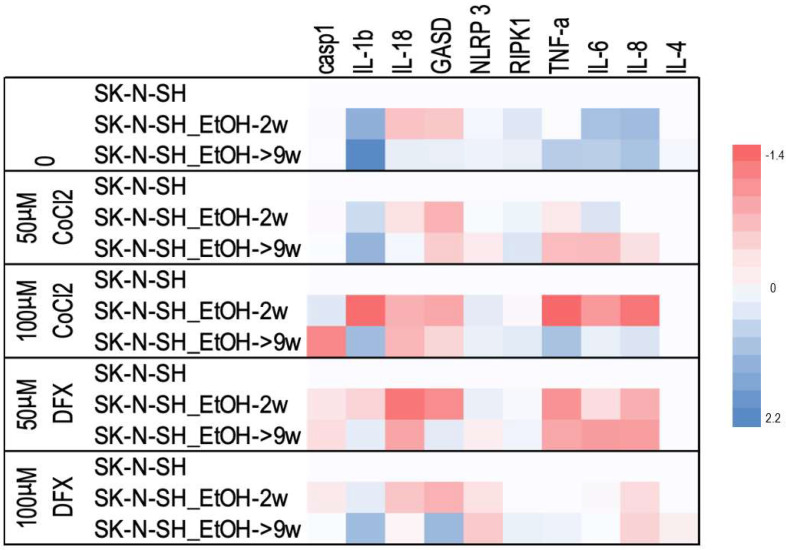
The effects of ethanol and hypoxic treatment on the gene expressions involved in pyroptosis and inflammation. Each numerical data was graphically represented using warm colors representing low-value data points and cool colours representing high-value data points.

The mRNA expression of nuclear factor kB (NF-kB) (Figure 7), another major transcription factor activated under stress by hypoxia or decreased oxygen availability [27], has been studied in cells treated with hypoxic agents. NF-kB mRNA expression was inhibited by chronic ethanol exposure and treatment with hypoxia mimetics in SK-N-SH cells. In addition, the NF-kB1 protein (Figure 9), which activates the proinflammatory cytokine genes [28], decreased in SK-N-SH cells chronically treated with ethanol with a similar trend after the addition of hypoxic agents. TGF-β mRNA expression (an NF-κB inhibitor) was stimulated in all hypoxia variants of the treatment investigated, except for SK-N-SH cells chronically exposed to ethanol and treated with 50 µM CoCl_2_. The same aspect was observed for the PCDH12 gene (involved in the stimulation of synaptic construction in the nervous system).

We observe similar dynamics in the protein synthesis of HSP70 and HSP60, being decreased in SK-N-SH cells chronically exposed to ethanol, even under additional hypoxemic conditions (produced under the action of DFX 100 µM and CoCl_2_ 100 µM). The synthesis and phosphorylation of HSP27 were stimulated in SK-N-SH cultures chronically exposed to ethanol, a situation that was also observed after treatment with DFX 100 µM (without being present also in the case of exposure to CoCl_2_ 100 µM, when synthesis and phosphorylation decreased HSP27); a fact that could show a cytoprotective effect of chronic ethanol exposure of neural cells, which seems to be amplified under the action of deferoxamine.

The dynamics of thioredoxin-1 (decrease in SK-N-SH cultures chronically exposed to ethanol, including in hypoxic conditions induced by treatment with DFX 100 µM and CoCl_2_ 100 µM) shows the antiapoptotic effect produced by chronic ethanol treatment, which diminishes (but remains) and under superfused hypoxic conditions. The behavior of thioredoxin-1 may show a neuroprotective effect of ethanol against oxidative stress. Sirtuin 2 was decreased in both chronically ethanol-treated SK-N-SH cultures and hypoxic stress overload by treatment with 100 µM deferoxamine and 2100 µM CoCl, which could result in more the efficient growth of neuronal terminals in cells chronically exposed to ethanol. Superoxide dismutase 2 (SOD2) protein synthesis is decreased in SK-N-SH cultures chronically treated with ethanol, a trend slightly increased under hypoxic conditions supplemented by treatment with 100 µM deferoxamine and 100 µM CoCl_2_. The variation of superoxide dismutase 2 may show how oxidative stress is decreased in the case of chronic ethanol treatment, increasing slightly in hypoxemic conditions (also found in SCI).

Protein synthesis of the transcription factor JNK activated by phosphorylation is inhibited in SK-N-SH cultures chronically treated with ethanol, showing a possible effect of decreasing axonal retraction (through dieback phenomena) under traumatic conditions. The overabundance of hypoxic stress (induced by DFX 100 µM and CoCl_2_ 100 µM) determined the increase in the synthesis of this cellular factor, probably as a result of cellular adaptation phenomena in the face of hypoxic stress phenomena.

The decrease in COX-2 synthesis may show a potential anti-inflammatory effect in SK-N-SH cultures chronically exposed to ethanol treatment. Synthesis of p-38α protein activated by phosphorylation decreased in the case of chronic ethanol treatment of SK-N-SH cells, a trend that was also maintained in the case of experimental hypoxic conditions achieved by additional treatment with deferoxamine 100 µM and CoCl_2_ 100 µM. The behavior of p-38α protein may show a protective effect against neurotoxicity achieved by chronic ethanol exposure and maintained even upon the addition of additional hypoxic stress. NF-kB1 protein synthesis (a translational factor involved in inflammation) decreased in SK-N-SH cultures chronically treated with ethanol, a trend also preserved in case of the addition of hypoxic conditions produced by treatment with CoCl_2_ 100 µM and deferoxamine 100 µM. The evaluation of NF-kB1 shows a possible anti-inflammatory effect of chronic ethanol exposure, an effect that is preserved under additional hypoxic stress conditions. The protein synthesis of antioxidant and anti-inflammatory molecules PON1 and PON3, which decreased in SK-N-SH cultures chronically treated with ethyl alcohol, was analyzed. Experimental hypoxic conditions improved PON values after treatment with deferoxamine 100 µM and cobalt chloride 100 µM. PON1 and PON3 variation shows how chronic ethanol treatment confers an anti-inflammatory and antioxidant effect on neural cells exposed to additional hypoxic conditions.

### 2.7. Analysis of Inflammatory Effects Induced by DFX and CoCl_2_ on Ethanol-Exposed Neural Cells

The minimal fluctuations of caspase-1 expression level were observed in all experimental points. The increase in IL-1β expression, especially in the case of long-term treatment with ethanol with and without additional hypoxic stress, was noted (Figure 9). In addition, the level of Gasdermin (GASD) has been increased in the chronic treatment with alcohol in the presence of DFX for 24 h.

In addition, the mRNA expression of a series of cytokines that could be affected by the presence of both ethanol and hypoxic factors was analyzed. Long-term ethanol treatment increased mRNA expression of pro-inflammatory cytokines (IL-1β, TNF-α, IL-6, and IL-8). It seems that in the presence of the hypoxia-inducing agents, only IL-1β stayed increased. One exception is the case of SK-N-SH EtOH-9w treated with 100 µM CoCl_2_, which also has high levels of TNF-α, IL-6, and IL-8 compared to cells non-exposed to ethanol.

### 2.8. Evaluation of Cell Recovery after Hypoxic Treatment Using the Scratch Assay

**Figure 10:**
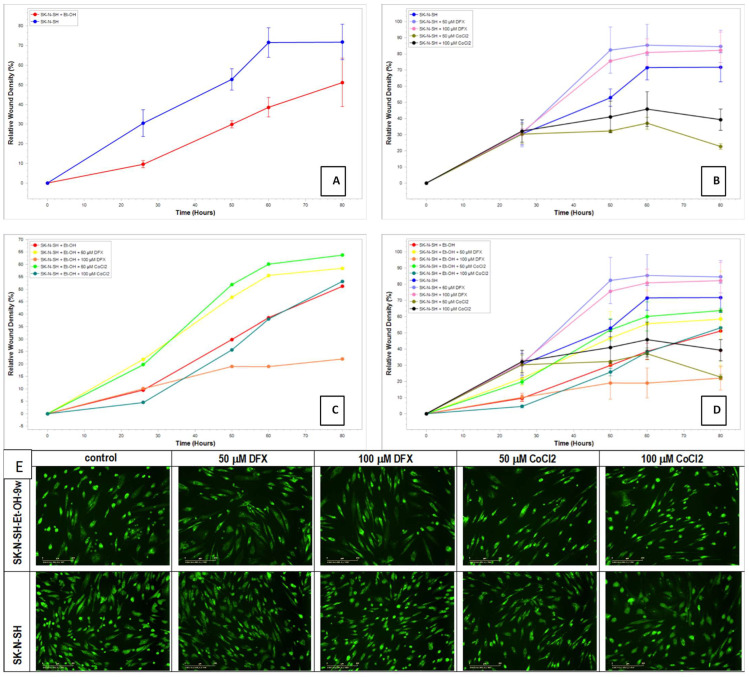
Summary data time courses of migration/wound healing of treated and untreated SK-N-SH cells; (**A**) SK-N-SH cells exposed or non-exposed at EtOH 50 mM for 9 weeks; (**B**) SK-N-SH cells treated post-wounding with 50 µM, respective 100 µM, DFX or CoCl_2_ for 24 h; (**C**) SK-N-SH cells grown in media with 50 mM EtOH for 9 weeks and treated post-wounding with 50 µM, respective 100 µM, DFX or CoCl_2_ for 24 h; (**D**) SK-N-SH cells grown in media without/with 50 mM EtOH for 9 weeks and treated post-wounding with 50 µM, respective 100 µM, DFX or CoCl_2_ for 24 h; (**E**) Acridine Orange/Propidium Iodide Stain (fluorescence, 10× objective).

The scratch/wounds assay evaluated the cell recovery potential after hypoxic treatment. The homogenous wounds were generated in ethanol-exposed or non-exposed SK-N-SH cells, and after that, the hypoxic treatment was applied for 24 h. The medium was changed with new fresh media (without hypoxic agents) for the remaining days of the experiment. Summary data time courses of migration/wound healing of treated and untreated SK-N-SH cells analyzed using Incucyte^®^ Scratch Wound Analysis Software Module are presented in Figure 10.

Regarding the migration–proliferation phase related to wound healing, the results showed that the ethanol non-exposed cells had a better proliferation than SK-N-SH_EtOH-9w EtOH (Figure 10A). DFX-treated cells displayed higher proliferative activity than the control cells while the treatment with CoCl_2_ decreased the proliferation–migration process (Figure 10B). The induction of hypoxia determined an active process of proliferation and migration, which helped heal the wound. The long-term exposure to EtOH 50 mM for 9w and the presence of hypoxic agents (Figure 10C) showed that the migration–proliferation had been more dependent on the concentration of the hypoxic agent than on the differences in the mechanism of hypoxic induction since at concentrations of 50 µM for both DFX and CoCl_2_, there is an increase in the proliferation–migration process compared to the cells not treated with hypoxic agents. The relative wound density was similar to the control cells.

In addition, the staining of cells with acridine orange/propidium iodide showed that all cells were viable (Figure 10E). The cells treated with 50 mM EtOH for 9 weeks had a decreased proliferation. On the other hand, the cells treated with EtOH and hypoxia agents maintained their cell density and seem morphologically in a better shape compared with untreated control cells. In comparison, cells not exposed to ethanol and treated with hypoxia agents are morphologically more affected than control cells not exposed to ethanol and hypoxia agents.

## 3. Discussion

Oxygen deprivation is a significant contributor to neurological conditions, such as spinal cord injury [29]. The changes produced by vertebral-medullary trauma are initiated by the suffering of neural cells produced by traumatic and vascular (ischemic and hemorrhagic) injuries, followed by local hypoxia [30]. For these reasons, we used the induction of cell hypoxia in cell cultures as a model to mimic the detrimental conditions detected in SCI but at the cell cultures level and not at the organ/systemic level, for which there are described experimental SCI models [31]. Our experimental model is original in explaining the clinical paradox observed through the positive correlation between chronic ethanol consumption and the relatively favorable evolution after SCI, as we showed in a previously published article [10].

Our work focused on the correlation between neural cells exposed in vitro to ethanol and, after, to hypoxic conditions as a model of cellular events that might occur after spinal cord injury in patients with chronic ethanol consumption. The ethanol effects were intensely studied previously. It was noted that if 12 mM blood alcohol levels induce anxiolytic and euphoric effects [32], increasing ethanol concentration induces sedation with coma or death at a concentration above 50 mM [33]. However, adaptation increases the alcohol tolerance to eight-fold higher in alcoholic persons compared with ethanol-naive persons [34,35].

On the other hand, it was reported that alcohol exposure retarded neural stem cell migration, neuronal formation, and growth processes by the alteration of the methylation process of specific genes associated with neural development [36]. Alcohol exposure inhibits methionine synthase and methionine adenosyltransferase, interrupting methylation processes [37,38]. Thus, alcohol exposure inhibits folate-mediated methionine synthesis and redirects folate utilization toward serine synthesis, interfering with methylene tetrahydrofolate and thymidine synthesis [37,38]. Due to folate deficiency, the misincorporation of uracil for thymidine during DNA synthesis increases the chromosomal breaks [39]. The ethanol toxicity is due to ethanol itself and its secondary metabolites such as acetaldehyde, NADH, acetylCoA, FAEEs, and ethanol ester Pet [35]. Ethanol interacts directly or indirectly with GABAA, glycine receptors, N-terminal and transmembrane 3 domains of NMDA receptors, protein kinase C, adenylate cyclase, and other many molecules [40,41], inducing a myriad of negative effects. The ethanol metabolite, PEt, induces changes in membrane polarization by competition with phosphatidic acid and phosphatidylinositol bisphosphate and inhibition of K^+^ ion channels [42]. Acetaldehyde, a transient toxic intermediate, can modify the structure and function of proteins, saccharides, and nucleic acid by their reactive carbonyl group that can bind compounds with amino, hydroxyl, and sulfhydryl groups [35,43]. The blood–brain barrier is not permeable for acetaldehyde [44], so, this is a more potent toxin than ethanol [35]. NADH, the by-product of ethanol metabolization, induces lactate production, and metabolic acidosis increases with a decrease in hemoglobin oxygen saturation. AcetylCoA, the final product of ethanol oxidation, amplifies ketogenesis [35].

In the developing CNS, ethanol-induced damage was observed through a mechanism involving apoptosis, accompanied by a decrease in the pAkt pro-survival pathway activity and reduction of pro-survival/antiapoptotic proteins controlled by NF-kB [45], such as Bcl-2, Bcl-XL, XIAP, cIAP-1, and cIAP-2. The generation of oxidative stress is also an important mechanism of ethanol-induced damage [46,47,48]. However, alcoholics do not generally consume pure alcohol; the drinks contain several other components, some of them with protective effects. Antonio & Druse investigated the potential neuroprotective effects of some antioxidants, such as resveratrol, curcumin, epigallocatechin-3-gallate, melatonin, and α-lipoic acid [45], which in addition to their antioxidant/free radical chelating effects, can activate a pro-survival pathway (α-lipoic acid, curcumin, and EGCG) [45,49,50,51] or can down-regulate the expression of several pro-apoptotic genes (EGCG) [52]. The studies of Antonio & Druse demonstrated that the co-treatment of fetal rhombencephalic neurons with 75 mM ethanol and different antioxidants prevents ethanol-associated apoptosis.

The nocive effects of alcohol consumption are already known, and we aimed to observe what happens when another stress is added, such as in an SCI. We chose to use the SK-N-SH cells (HTB-11) neural cells to evaluate the specific effects of hypoxia-inducing agents on ethanol treated cells. Once the ethanol and hypoxemic agents working concentrations were set, the molecular mechanisms were evaluated. The Incucyte test highlights the cell population’s recovery after a hypoxic agent’s action validating that both non-alcoholic and alcoholic cells recover after stress removal. We chose two concentrations and two chemical inductors of hypoxic stress to assure the control of the exposure doses of well-known hypoxic inductors. Our study shows that two markers of intracellular hypoxia, proteins HIF-1α and HIF-2α [26], were inhibited in the presence of alcohol and increased by treatment with hypoxic agents. Under normal oxygenation, HIF-1 protein is degraded by ubiquitinylation. This process is inhibited under hypoxic conditions, allowing HIF-1 to activate genes containing hypoxic response elements (HREs) in their structure [53]. Some HIF-1α target genes have potential neuroprotective roles (like VEGF and GLUT-1). Several other proteins play roles in ROS processes, such as CA IX (involved in controlling cellular pH [54], neutralizing ROS [55]), Phospho-JNK (stimulating intracellular nitric synthetase [56], and PON1 (a molecule with antioxidant effect [57]) were stimulated in the presence of hypoxemic agents, while the chronic treatment with ethyl alcohol had determined their inhibition.

Moreover, hypoxic stress might cause cellular energy depletion directly proportional to its intensity due to a decreasing tendency of AMP phosphorylation (proportional to the molar concentration). It was noted that hypoxic stress seems to amplify energy production through aerobic glycolysis [58], and our results sustain it since an increase in PGK1 values was observed in SK-N-SH cells treated chronically with ethanol and hypoxic stressors. In addition to aerobic glycolysis, the activation of the anaerobic pathways depends on the treatment. We found that PFKL1 gene expression was inhibited by chronic ethanol treatment and stimulated under hypoxic stress. LDH B gene expression was stimulated in chronic ethanol treatment and inhibited by hypoxic stress. Most likely, there is a tendency to increase the production of lactic acid by decreasing its dehydrogenation to pyruvic acid [59].

Additionally, the levels of Claspin protein were increased in neural cells maintained in the presence of ethanol. Claspin is an unstable protein, as it is degraded by ubiquitination and controlled by numerous ubiquitin ligases and deubiquitinases, with varying levels throughout the cell cycle (because it can bind directly to DNA and interact with many protein components of the replisome, which is required to maintain normal rates of progression of DNA replication). Claspin is also involved in neoplastic progression or suppression of DNA synthesis in response to non-genotoxic stress in the endoplasmic reticulum [60]. Clusterin has a protective role against apoptosis mediated by endoplasmic reticulum stress or oxidative stress [61]. Clusterin is a highly sialylated glycoprotein that needs the addition of sugars to the molecule for maturation. In alcoholics, sialyltransferase activity may be reduced, decreasing the incorporation of sialic acid molecules in the mature clusterin [62]. Our experiments showed a decreased clusterin in the presence of alcohol, which might be correlated with decreased protection from stress. Cellular stress is also an activator of p53, a short-lived transcription factor [63]. P53 coordinates the growth status of the cell [64] through a flexible program inducting either cell-cycle arrest (transient or permanent) or cell death [65]. The molecular mechanisms that discriminate between distinct responses of a plethora of p53 functions are based on post-translational modifications [66]. S46 phosphorylation promotes transactivation of pro-apoptotic target genes and enhances p53 mitochondrial translocation [66]. Ser-15 phosphorylation of p53 is up-regulated during cellular stress [67]. Serine 392 phosphorylation modulates p53 mitochondrial translocation and transcription-independent apoptosis [68]. DFX is responsible for the p53 phosphorylation (Figure 6) by EtOH, which is associated with the increased cell growth observed in SK-N-SH-EtOH-9w (Figure 10), sustaining a possible neuroprotection.

The molecular mechanisms of neural cell death are complex and are accomplished through several well-regulated pathways. In spinal cord injury, the changes are initiated by the distress and death of neuronal cells caused by traumatic and vascular damage (ischemic and hemorrhagic), followed by hypoxic stress (diminution or deprivation of oxygen in organs, tissues, and cells). Inflammasome, a multiprotein cytosolic complex that functions as an intracellular receptor for cellular and environmental stress [69], mediates the innate immune response, which can cause tissue damage when it is over-activated [70,71]. Inflammation complexes in the central nervous system are in the pre-assembled state before they are activated, which causes the rapid post-traumatic activation of the innate immune response [70]. Inflammation-mediated neuronal death, pyroptosis, consists of disruption of the neuronal membrane’s integrity by forming membrane pores, followed by osmotic cell swelling [72]. As a result of pyroptosis, the components of the inflammatory complex are eliminated in the intercellular space, where they amplify tissue inflammation [70]. Abnormal activations of the inflammasome occur in traumatic lesions of the central nervous system [70]. In the case of spinal cord injuries, inflammasome activation mediates the inflammatory response through IL-18 and IL-1β with the activation of T helper lymphocytes (LTh1). The excessive production of IL-18 and IL-1β can disrupt the continuity of the blood–neural axis and the production of neurotoxic molecules, with secondary neuronal death in SCI. The mechanism of NLPR3 receptor activation (which belongs to the host’s innate immune response and inflammatory response) in SCI is not understood. Still, it is thought that it could be the action of extracellular adenosine triphosphate (ATP), the generation of mitochondrial reactive oxygen species (ROS), and the decrease of intracellular potassium below 90 mM [70].

Thus, another point evaluated by us was pyroptosis (or caspase 1-dependent cell death), a lytic and inflammatory form of cell death activated by microbes [73], and endogenous damage (including cytosolic double-stranded DNA (dsDNA), crystals and toxins) [74]. It was noted that chemotherapeutical drugs induce pyroptosis through mitochondrial death machinery, and caspase 3 cleaves GASD. In turn, GASD activates the NLRP3 inflammasome, leading to the activation of caspase 1/GASD cascade, which promotes the maturation of IL-1β and IL-18 [73].

We observed minimal fluctuations regarding caspase-1 expression levels in all experimental points. Of note was the increase in IL-1β expression, especially in the case of ethanol long-term treated cells with or without added hypoxic stress. Thus, the expression of the IL-1β gene shows the early inflammatory response after hypoxic stress production (following the activation of the inflammasome by the canonical pathway). In contrast, IL-18 (another cytokine-stimulated by inflammation) was rather inhibited by both ethanol and hypoxic agents. In addition, the level of GASD (which represents the common stage of the inflammasome pathways, canonical and non-canonical) has been increased in the chronic alcohol-treated cells together with DFX for 24 h. This means that chronic ethanol consumption might activate inflammation and potentiate the effects induced by some chemotherapeutics.

TNF-α cytokine expression (which activates the extrinsic apoptotic pathway and inflammation) was increased by chronic ethanol treatment (9w) and high concentrations of hypoxemic agents (100 µM). In contrast, in the alcohol 2w treatment in the presence of low concentrations of hypoxemic agents, TNF-α expression was inhibited, supporting the decrease of inflammation. The same profile was observed for IL-8 and IL-6. Only long-term treatment with ethanol shows minimal activation of IL-4 expression, a cytokine involved in inhibiting inflammation.

It was our assumption, confirmed by the presented results, that the viable cells after long-term ethanol exposure, acquired mechanisms of adaptation and resistance to cellular stress. However, in hypoxic shock-induced trauma (DFX or CoCl_2_) this effect is not supported, at least in the acute phase (24-h treatment with hypoxic agents).

Many cellular mechanisms are influenced by ethanol consumption, such as intracellular signaling, ROS generation, apoptotic cascades, intracellular ionic homeostasis. Removing damaged mitochondria with autophagy sustains cell survival, and there are findings that support the protective role of autophagy in ethanol and hypoxic conditions [75].

## 4. Materials and Methods

Cell lines—SK-N-SH: Human Neuroblastoma Cell Line (ATCC ATCC HTB-11, Rockville, MD, USA) were maintained in Dulbecco’s Modified Essential Medium, DMEM: F12 (Sigma-Aldrich, St. Louis, MO, USA) supplemented with 10% inactivated fetal bovine serum (FBS) (Sigma-Aldrich, USA), 100 units/mL penicillin and 0.1 mg/mL streptomycin (Sigma-Aldrich, USA). The cells were kept in a humidified atmosphere at 37 °C containing 5% CO_2_.

Ethanol Cell treatments—For ethanol treatments, the cells were seeded in 25 cm^2^ flasks at a cell density of 1 × 10^6^. Ethanol (Merck, Germany) was diluted in DMEM: F12 containing 10% FBS. To establish the ethanol concentration for long-term treatment, the following ethanol concentrations were tested: 50 mM, 100 mM, 200 mM, and 300 mM. The medium was changed daily for 7 days of treatment. To study the effect of long-term ethanol exposure, the SK-N-SH cells were treated daily with a fresh medium containing 50 mM ethanol for 2 weeks (w). To mimic the effects of chronic ethanol consumption, the cells were exposed over 9 weeks to ethanol by daily replacement with a fresh medium containing 50 mM ethanol. SK-N-SH cells exposed 2 weeks to ethanol will be referred to as SK-N-SH_EtOH-2w; SK-N-SH cells exposed over 9 weeks to ethanol will be referred to as SK-N-SH_EtOH-9w.

Hypoxic agents treatments—To generate hypoxic conditions, cells were treated with a hypoxia inducer, Desferal/Deferoxamine (DFX) (Novartis, Nuernberg, Germany) or cobalt chloride (CoCl_2_) (Merck, Darmstadt, Germany) for 24 h at a concentration of 50 µM or 100 µM.

Cell viability—The viability of the cells treated with increasing ethanol concentrations was determined by Trypan Blue exclusion. The live cells (negative for staining) and dead cells (positive for staining) were counted using a Burker-Turk hemocytometer.

To assess the viability of ethanol-treated cells in the presence of hypoxia inducers, CellTiter-Glo luminescent assay (G7570, Promega, Madison, WI, USA) was used. Nontreated cells, two and over nine weeks ethanol-treated cells were plated in triplicates onto opaque 96-well plates for 24 h. Then, cells were treated with either DFX or CoCl_2_ as described above for either 6 or 24 h. Following the treatment, cells were incubated for 10 min with CellTiter-Glo reagent, and luminescence was measured using a 96-well plate reader (BertholdTech TriStar2S 96 microplate luminometer; Promega). Background luminescence was measured in a medium without cells and subtracted from experimental values.

Reactive oxygen species generation—Generation of ROS was detected using the luminescent ROS-Glo H_2_O_2_ assay (Promega, USA). Nontreated cells, cells treated with ethanol for two weeks, and cells treated with ethanol over nine weeks were plated in Nunc F96 microwell white plates (ThermoFisher Scientific, USA) at a density of 7.5 × 10^3^ per well in 80 μL DMEM as recommended by the manufacturer. After the cells were attached, they were treated with 50 µM or 100 µM hypoxia inducers for 6 or 24 h. After this period, substrate solution was added to a final concentration of 25 μmol/mL, and cells were incubated for 6 h. Then, 100 μL ROS-Glo detection solution was added to each well for 20 min at room temperature. Relative luminescence units were recorded using the microplate reader. The experiment was run in triplicates.

Gene expression analysis by qRT-PCR—Cells treated with ethanol for 2 or 9 weeks followed by 24 h treatment with hypoxic inductors were homogenized in Trizol following the manufacturer protocol for RNA isolation. The RNA quality and quantity was assessed for all samples using NanoDrop (Thermo Fisher Scientific, Waltham, MA, USA). cDNA synthesis was performed using High-Capacity cDNA Reverse Transcription Kit (Thermo Fisher Scientific Inc., USA) according to the manufacturer’s protocol. The gene expression analysis was performed using taqman assays (Thermo Fisher Scientific, USA) for the following genes: CDH2 (Hs00169953_m1), Casp3 (Hs00234387_m1), Casp8 (Hs00154256_m1), Casp7 (Hs00169152_m1), Casp1 (Hs00354836_m1), Casp9 (Hs00154261_m1), MCL1 (Hs00172036_m1), BCL2 (Hs00608023_m1), Bax (Hs00180269_m1), CD133 (Hs01009256_m1); SHH (Hs01123832_m1), NOTCH1 (Hs01062014_m1), NFKB1 (Hs00765730_m1), Hey1 (Hs01114113_m1), GFAP (Hs00157674_m1), BMP4 (Hs00370078_m1), Hes1 (Hs00172878_m1), NEUROD1 (Hs00159598_m1), WNT1 (Hs01011247_m1), OLIG2 (Hs00377820_m1), TNF (Hs00174128_m1), Nes (Hs007070120_m1), Vim (Hs00185584_m1), TGFB1 (Hs00998130_m1), IL-18 (Hs01038788_m1), IL-4 (Hs00174122_m1), IL-1β (Hs01555410_m1), IL-2 (Hs00174114_m1), IL-6 (Hs00174131_m1), IL-10 (Hs00174086_m1), TIMP3 (Hs00165949_m1), VEGF-a (Hs00900055_m1), HIF1 (Hs00153153_m1), PCDH12 (Hs00170986_m1), and FLT1 (VEGFR) (Hs01052961_m1) and using qPCR primer pairs (OriGene Technologies, Inc., Rockville, USA) for the following genes: Casp7 (CAT#: HP233752), Casp9 (CAT#: HP205155), Casp8 (CAT#: HP234494), Casp10 (CAT#: HP228052), GasderminD (CAT#: HP214995), RIPK1(CAT#: HP207257), TNF-α (CAT#: HP200561), PON2 (CAT#: HP200286), GLUT1 (CAT#: HP209446), LDH-B (CAT#: HP206024), ALDO-A (CAT#: HP200021), AK-3 (CAT#: HP212098), PGK-1-F (CAT#: HP200272), PFK-L-F (CAT#: HP228792), Tie-1-F (CAT#: HP200439), TIMP-1-F (CAT#: HP206804), HMGB-1 (CAT#: HP205869). The relative fold differences in gene expression were calculated based on ∆∆Cq method. The amount of reverse-transcribed RNA was 2 µg/reaction. The qRT-PCR program included 43 cycles.

Protein Array Analysis—Cell lysates prepared from SK-N-SH cells (2 w or 9 w), treated or untreated with hypoxic inductors (for 24 h), were analyzed for apoptosis-related proteins using Proteom Profiler Human Apoptosis Array (ARY 009, R&D Systems Minneapolis, MN, USA) and Proteome Profiler Human Stress array kit (ARY 018, R&D systems, Minneapolis, MN, USA) according to the manufacturer’s instructions. Briefly, cell lysates were prepared and protein concentration was measured using the Bradford protein assay kit (PierceTM, Thermo Scientific, Waltham, MA, USA). Cell lysates (400 µg for apoptosis array ARY 009 and 100 µg for stress array ARY 018) were diluted in array buffer and incubated with the ready-to-use pre-coated array membranes overnight at 4 °C on a shaker. The membranes were washed three times to remove any unbound proteins and then incubated with the detection antibody cocktail for 2 h on a shaker at room temperature. After washing, the membranes were further incubated with diluted streptavidin-HRP for 30 min at room temperature. Chemi Reagent mix was added to detect the protein spots by chemiluminescence. The spots were visualized, and images were captured using MicroChemi 4.2 (DNR Bio-Imaging System Ltd., Jerusalem, Israel). The densitometric analysis of the protein spots was performed using ImageJ 1.52a software. The pixel density of each duplicated protein spot from the array was averaged and normalized against the kit’s reference spots, and the relative levels were expressed as mean pixel intensity.

Scratch assay—Ethanol-exposed and non-exposed SK-N-SH cells were seeded at a concentration of 10^4^ cells per well on a 96-well ImageLock tissue culture plate (Essen BioScience 4379) and incubated at 37 °C in a humid atmosphere for 24 h. Wound Maker, Incucyte^®^ Woundmaker Tool (Cat. No. 4563) was used to generate homogenous wounds in the cell monolayer according to the manufacturer’s protocol. After wounding, the medium was removed, the cells were washed twice, and 100 µL culture medium (DMEM + F12 + 10% FBS) containing 50 µM or 100 µM, DFX or CoCl_2_ was added. After 24 h, the medium was removed, new fresh medium (without hypoxic agents) was added, and the plate was placed in the IncuCyte System. Scanning was scheduled every 2 h for 96 h in the Incucyte^®^ Scratch Wound Analysis Software Module (Cat. No. 9600-0012). After the first scan, the initial scratch wound mask used in the subsequent analysis was created. The relative wound density (RWD) relies on the initial scratch wound mask defining the initial wound region generated based on the first images collected from each well. The relative wound density is a measure of the wound region density relative to the density of the cell region: %RWD(t) = 100 × (w(t) − w(0))/(c(t) − w(0)); where: w(t) = density of wound region at time (t); c(t) = density of cell region at time (t).

Acridine Orange/Propidium Iodide Stain—The cells were double-stained with acridine orange (AO) and PI to evaluate cell recovery after hypoxic treatment and the scratch assay. The dyes (10 µg/mL AO and 10 µg/mL PI) were added, and after 15 min incubation, the medium was removed. The cells were washed with PBS, and the images were taken using the Incucyte system. Both acridine orange and propidium iodide are fluorescent dyes that bind to nucleic acids. The difference is that acridine orange can pass through the intact cell membrane, while propidium iodide can only stain nucleic acids in the cells with a compromised membrane. Therefore, this staining is able to discriminate between viable cells emitting green fluorescence and non-viable red fluorescent cells.

Statistical analysis—Statistical analyses were performed using an unpaired Student’s *t*-test. A *p*-value < 0.05 was considered statistically significant, * *p* < 0.05, ** *p* < 0.005.

## 5. Conclusions

The study was performed to evaluate the responses of long-term ethanol exposed SK-N-SH cells to hypoxia agents. This model could mimic changes that occurred in alcoholic patients having spinal cord injury and provide a better understanding of the cellular events. The long-term presence of ethanol changed the expression pattern of the investigated molecules. Some of our results show that long-term ethanol exposure has a cytotoxic effect inducing apoptosis that is consistent with clinically observed neurodegeneration in chronic ethanol-consuming patients. A 24 h exposure to hypoxic stress (deferoxamine or cobalt chloride) reduced ROS in long-term ethanol-exposed SK-N-SH cells, which might be due to an adaptation to stressful conditions. HIF-1α protein level was increased after hypoxic treatment in long-term ethanol-exposed cells, inducing fluctuations in its target metabolic enzymes proportionally with treatment intensity. At least in the acute phase (24-h treatment with hypoxemic agents), hypoxic shock increased IL-1 and TNF- α in chronic ethanol-treated cells. However, the cells recovered after stress conditions. The wound-healing assay showed that the ethanol-exposed cells that passed the acute stress had the same proliferation profile as non-ethanol exposed cells. DFX-treated cells displayed higher proliferative activity than the control cells in the proliferation-migration process, supporting the neuroprotective effect. The cells have exceeded the critical moment of the ethanol-induced traumatic effects and display an adaptive mechanism to ethanol (chronic phenomenon), allowing them to sustain regeneration. The cellular response in terms of proliferation and migration coupled with the molecular response showed that when the second cellular stress (induced hypoxia) is added, cells exposed to ethanol for a long time have a lower degree of hypoxia damage; however, further studies are needed to confirm these results. These studies need to be deepened and future experiments conducted to respond to many questions: (1) after the stress removal, which are the molecular mechanisms involved in cellular recovery; (2) what are the differences between ethanol exposed and non-exposed cells during recovery; (3) what is the microenvironment contribution to the recovery process; (4) do the ethanol exposed cells have a higher recovery potential or better adaptability to stress than non-exposed cells?

## Data Availability

Not applicable.
